# Impact of Blood Pressure After Successful Endovascular Therapy for Anterior Acute Ischemic Stroke: A Systematic Review

**DOI:** 10.3389/fneur.2020.573382

**Published:** 2020-10-29

**Authors:** Benjamin Maïer, François Delvoye, Julien Labreuche, Simon Escalard, Jean-Philippe Desilles, Hocine Redjem, Solène Hébert, Stanislas Smajda, Gabriele Ciccio, Bertrand Lapergue, Raphaël Blanc, Michel Piotin, Mikael Mazighi

**Affiliations:** ^1^Interventional Neuroradiology Department, Fondation Rothschild, Paris, France; ^2^Université de Paris, Paris, France; ^3^University Lille, CHU Lille, EA 2694 - Santé publique: épidémiologie et qualité des soins, Lille, France; ^4^Laboratory of Vascular Translational Science, INSERM U1148, Paris, France; ^5^Stroke Center, Foch Hospital, Suresnes, France; ^6^FHU Neurovasc, Paris, France

**Keywords:** blood pressure, ischemic stroke, endovascular treatment (EVT), intracranial heamodynamics, disability, reperfusion after ischemia

## Abstract

**Background and Purpose:** Optimal blood pressure (BP) targets after endovascular therapy (EVT) for acute ischemic stroke (AIS) still need to be assessed, especially according to the recanalization status. Facing the lack of randomized controlled trials addressing this question, we performed a systematic review of studies assessing the post-EVT BP impact on functional outcome and symptomatic intracranial hemorrhage (sICH).

**Methods:** Studies published after January 1, 2012 were included in the systematic review. The PRISMA checklist and flow diagram were followed for the design and reporting of this work.

**Results:** Five studies were included in the present analysis. Despite a significant heterogeneity among studies which precluded a meta-analysis, systolic BP (SBP) was the most frequently used parameter to describe BP. BP variability (standard deviation, successive variability) after EVT was associated with worse functional outcome, especially in studies without specific BP targets after successful EVT. Lower BP values after successful EVT were associated with lower odds of sICH. Four studies evaluated the post-EVT BP impact on recanalized patients solely, with only one specifically addressing the impact of a TICI 2B vs. 2C. Interestingly, SBP reduction was inversely associated with worse outcomes in TICI 3 patients but not in TICI 2B patients, pointing to the potential value of BP management according to the exact TICI.

**Conclusions:** BP post-EVT seems to be associated with worse functional outcomes and sICH. However, given the important heterogeneity depicted among the included studies, no decisive conclusion can be made from this systematic review, thus underlying the urgent need of randomized controlled trials evaluating this question.

## Introduction

With reperfusion rates currently exceeding 90% at the end of procedure (1), endovascular therapy (EVT) is now the reference treatment of acute ischemic strokes (AIS) with an anterior large vessel occlusion (LVO) (2). However, 3-month functional outcomes still remain poor with almost 50% of recanalized patients having an unfavorable outcome (3). Among the several reasons for this result, recent lines of evidence have shed light on the role of blood pressure (BP) management before (4), during (5), and after EVT (6–9). In contrast to the BP management of patients exclusively treated by intravenous thrombolysis, the hemodynamic management of LVO treated by EVT could be intuitively seen as bi-phasic. Simply stated, the pre-reperfusion phase would thus prevent hypotension in order to avoid any extension of the ischemic penumbra; (10) while the post-reperfusion phase could prevent reperfusion lesions or symptomatic intracranial hemorrhage (sICH) in case of reperfusion, (6, 11) or extensive infarct growth in case of incomplete reperfusion (12). That said, current international guidelines for BP management during and after EVT do not seem to discriminate BP management between the two treatments situations, (2) mainly because no randomized controlled trial (RCT) has ever evaluated this strategy.

Numerous observational studies have recently outlined the association between higher post-reperfusion BP values and sICH or worse functional outcomes, notably according to the reperfusion status (6–9, 11, 13–25). A recent systematic review and meta-analysis focusing on mean systolic BP (SBP) and diastolic BP (DBP) suggested that increased BP after EVT was associated with sICH and 3-month functional dependence (26). Still, many questions remain unanswered such as the specific BP target to achieve, the best pharmacological approach according to the reperfusion status and the impact of BP variability after EVT. Therefore, we sought to perform an additional narrative systematic review of studies addressing the effect of BP after successful EVT on functional outcome and/or sICH. Here, we discuss in detail the impact of different BP parameters (steady vs. dynamic) after successful EVT on primary and safety outcomes, as well as the design of upcoming RCT in this area.

## Methods

The authors declare that all supporting data are available within the article (and its online Supplementary Files).

The Preferred Reporting Items for Systematic Reviews and Meta-Analyses (27) checklist and flow diagram were followed for the design and reporting of this work, as detailed below (27).

### Searching Strategy

Two independent authors (BM, FD) performed a systematic search on PubMed and Scopus database on May 3, 2020 to screen studies published in English after January 1, 2012. This date was chosen for two main reasons: (1) previous screening of the literature published prior to 2012 showed that EVT was mainly performed using intra-arterial tPA and with “older generation” devices, (2) SOLITAIRE FR (the main device used in randomized trials that showed benefit of EVT) was approved by the FDA in 2012. The search strategy developed for MEDLINE using MeSH terms and Scopus databases are detailed in Supplementary Table 1.

### Study Inclusion

We included studies that met the following criteria: (1) patients with AIS consecutive to anterior LVO; (2) >90% of the study patients treated with EVT (with or without intravenous thrombolysis) using first or second generation devices; (3) systematic BP monitoring after EVT; (4) sICH and/or functional outcome assessment at 3 months using the modified Rankin scale (mRS) according to BP data and (5) main analysis on successful reperfused patients (mTICI 2B or 3). Studies evaluating the impact of intraoperative hemorrhage on functional outcomes were excluded, as this topic is beyond the scope of this review. Finally, overlapping data (same patient data used in >1 publications) for the outcomes of interest were taken into accounts, owing to the narrative and exploratory nature of the present analysis.

### Data Collection and Extraction

Data were independently extracted from the selected articles by two readers (BM, FD) using a standardized case report form and discrepancies were solved by consensus. The items extracted are available in the Supplementary Material. Our search yielded to 11 different definitions of hemodynamic parameters which precluded any meta-analysis of association between hemodynamic parameters and outcomes (28).

## Results

### Study Selection and Description

**Figure 1:**
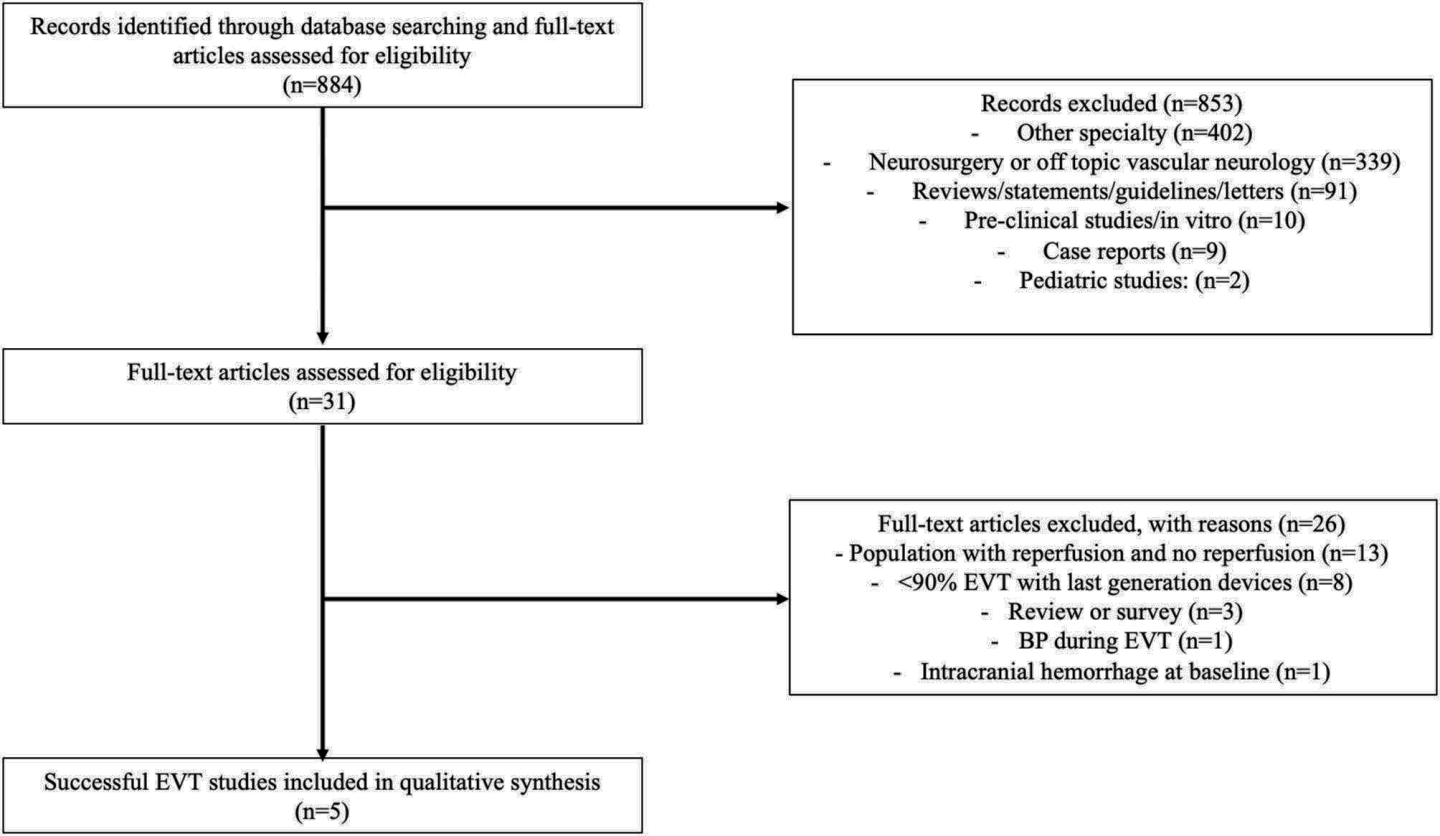
Flow chart presenting the research database, the screening process and the number of included studies with reasons of exclusion.

As illustrated in the flow chart (Figure 1) and in the Supplementary Methods, 18 studies evaluated BP impact after EVT (Supplementary Table 2) (6, 7, 9, 11, 13–19, 21–25, 29, 30). Among them, 5 studies were specifically designed to assess BP impact after successful reperfusion and were included for the present systematic review (Table 1, details in Supplementary Table 3) (6, 11, 13, 16, 30). All studies were retrospective, and four were multicentric.

**Table 1:** Characteristics of included studies with BP targets and BP modality of assessment.

**Author, year**	Citation Nb^*^	**Nb of patients**	**Hypertension (*N*, %)**	**Reperfusion rates**	**BP assessment**	**BP targets and measures**	**Hemodynamic treatments**
**Studies with specific post-EVT BP targets**							
Kim et al. (11)	8	211	124 (58.8%)	***Only successful reperfusion*** TICI 2B: *N* = 105 (49.8%) TICI 3: *N* = 106 (50.2%)	Non-invasive SBP and DBP per hour within 24 h post-EVT	***Target: SBP<180 mmHg*** Measures: Mean (average BP), maximum, minimum, maximum-minimum BP variability: SD^a^, SV^b^, CV^c^, BP fluctuation in relation to time (TR^d^)	Antihypertensive treatment: *N* = 100 (47.4%)
Chang et al. (16)	0	90	43 (49.4%)	***Only successful reperfusion*** TICI 2B: *N* = 50 (55.6%) TICI 3: *N* = 40 (44.4%)	Non-invasive Every 15 min for 2 h, every 30 min for 6 h, and hourly for the remaining 16 h post-EVT	***Target for patients with successful reperfusion: SBP<160 mmHg*** Threshold between SBP < 140 or 160 mmHg depended on the physician. *Measures:* Mean SBP ≤ 130 mmHg, mean SBP>130 mmHg, 24h mean SBP, percent reduction ≤ 15%, percent reduction >15%	Antihypertensive treatment: *N* = 39 (43.3%) Calcium channel blockers: *N* = 35 (89.7%) Beta blockers: *N* = 4 (10.3%) Angiotensin II receptor blockers: *N* = 2 (5.1%) Continuous IV infusion: *N* = 17 (43.6%)
Anadani et al. (13)	0	1019	644 (63.2%)	***Only successful reperfusion*** TICI 2B: *N* = 454 (44.6%) TICI 3: *N* = 565 (55.4%)	Invasive and non-invasive (according to centers) BP measured within the first 24 h No standard protocol for BP measurements	***Target: Intensive BP control (SBP < 140 mmHg); moderate BP control (SBP<160 mmHg); guideline-recommended BP control (SBP < 180 mmHg)***. Mean SBP: mean value of all available SBP during 24 h Relative SBP reduction during 24 h: (Admission SBP—Mean SBP)^*^100/admission SBP	Type and timing of antihypertensive treatments not available.
**Studies without specific post-EVT BP targets**							
Anadani et al. (13)	0	1361	854 (63%)	***Only recanalized patients*** TICI 2B: *N* = 632 (46.4%) TICI 3: *N* = 729 (54%)	Modality not mentioned SBP measured at baseline and during 24 h post-EVT	SBP reduction (SBPr)^g^, analysis according to baseline SBP ( ≤ 140 or >140 mmHg)	Anti-hypertensive treatment within 24 h: *N* = 549 (54%)
Anadani et al. (6)	4	1245	797 (64%)	***Only recanalized patients*** TICI 2B: *N* = 508 (41%) TICI 3: *N* = 738 (59%)	Non-invasive with BP cuff or arterial line if available BP measured hourly for at least 24 h post-EVT	SBP, DBP: mean, maximum, minimum SBP range: maximum-minimum SBP SD	Anti-hypertensive treatment: *N* = 571 (46%) Vasopressors: *N* = 111 (8%)

BP, blood pressure; EVT, endovascular therapy; IVT, intravenous thrombolysis; CI, contraindicated; Nb, number; SBP, systolic blood pressure; DBP, diastolic blood pressure; MAP, mean arterial pressure; HR, heart rhythm; ARV, average real variability.

a *SD, standard deviation of the mean*,

b *SV, successive variation*,

c *CV, coefficient of variation (SD/mean BP)*,

d TR, time rate.

g (aSBP-mSBP/aSBP) ×100 where aSBP is admission SBP and mSBP mean SBP during the 24 h after EVT.

* *Google scholar searched performed on May 3, 2020*.

### Hemodynamic Parameters and Treatments

#### Modality of BP Assessment, BP Targets and BP Variability After EVT

BP was monitored non-invasively after EVT in three studies, (6, 11, 16) both invasively and non-invasively according to the center in one study (30) and not mentioned in one study (13) (Table 1). Three studies proposed a BP target after EVT (11, 16, 30). Among them, one study proposed three BP targets: an intensive BP control (SBP < 140 mmHg), a moderate (SBP < 160 mmHg) and guideline-recommended BP control (SBP < 180 mmHg), (30) Table 1. The second study recommended an SBP < 180 mmHg after successful EVT (11) and the last one an SBP < 160 mmHg with thresholds between SBP < 140 or 160 mmHg according to the physician in charge (16).

Overall, SBP was the most studied parameter after EVT. Dynamic (BP variability) parameters consisted of standard deviation (SD), coefficient of variation (CV), successive variation (SV, being the square root of the average of squared difference between successive BP measures), BP range (maximum-minimum), SBP reduction ([admission SBP-mean SBP during the first 24 h/admission SBP] ×100) or BP fluctuation in relation to time (time rate: TR), Table 1. Steady BP consisted of mean (average) and time spent under different SBP threshold (mainly 130 or 140 mmHg).

### Relationship Between BP After Successful EVT and Functional Outcome

#### Studies With Specific Post-EVT BP Targets

Among the three studies with specific post-EVT BP targets, two specifically assessed BP parameters with functional outcome after successful EVT (16, 30). In these studies, steady BP parameters consisted of an intensive BP control (SBP < 140 mmHg), which was associated with higher odds of good functional outcome compared with guideline-recommended BP targets (SBP < 180 mmHg) (30), while mean SBP > 130 mmHg (OR = 2.66 [1.11–6.41]) (16) was associated with poor outcome, Table 2. No dynamic parameters (BP variability) were evaluated in these studies with strict post-EVT BP targets.

**Table 2:** BP parameters associated with functional outcome in multivariate analysis, in the overall population and according to the reperfusion status.

**BP parameters**	**Results (overall)**	**Results according to the reperfusion status**
**Studies with specific post-EVT BP targets**		
***Dynamic BP parameters (BP variability)***		
None		
***Steady BP parameters***		
**SBP** **<** **140 mmHg** (30)	OR = 1.53 (1.07–2.19)	***Higher odds of good functional outcome compared with guideline-recommended BP targets (SBP < 180 mmHg)*** Subanalysis with patients with pre-treatment SBP ≥ 140 mmHg: Intensive (SBP < 140 mmHg) and moderate (SBP < 160 mmHg) BP targets had higher odds of good functional outcome than guideline-recommended BP targets: OR = 1.75 (1.07–2.85) and OR = 2.30 (1.17–4.52), respectively.
**Mean SBP** **>** **130 mmHg** (16)	OR = 2.66 (1.11–6.41)	Only patients with successful reperfusion
**Studies without specific post-EVT BP targets**		
***Dynamic BP parameters (BP variability)***		
**rSBP** **(****13****)**	OR = 0.97 (0.95–0.98), *p* < 0.001	***Subgroup analysis in patients with TICI 3:*** OR = 0.98 (0.97 to 0.98), *p* = 0.014 ***Subgroup analysis in patients with TICI 2B:*** no significant results
**Maximum SBP** **(****6****)**	OR = 0.9 (0.85–0.95), *p* < 0.001	***Patients with TICI 3:*** aOR = 0.9 (0.84–0.97), *p* = 0.007 ***Patients with TICI2B:*** aOR = 0.9 (0.83–0.97), *p* = 0.010
**SBP range** **(****6****)**	OR = 0.91 (0.86–0.96), *p* = 0.003	***Patients with TICI3:*** aOR = 0.9 (0.84–0.97), *p* = 0.009 ***Patients with TICI2B:*** aOR = 0.92 (0.85–1), *p* = 0.037
***Steady BP parameters***		
**SBP Mean** **(****6****)**	OR = 0.86 (0.79–0.93), *p* < 0.001	***Patients with TICI 3:*** aOR = 0.85 (0.75–0.96), *p* = 0.008 ***Patients with TICI2B:*** aOR = 0.87 (0.77–0.99), *p* = 0.029

#### Studies Without Specific Post-EVT BP Targets

Two studies without specific post-EVT BP targets evaluated the association between dynamic BP parameters and functional outcome. In these studies, SBP reduction (OR = 0.97, [0.95–0.98]), (13) maximum SBP (OR = 0.90, [0.85–0.95]) (6) and SBP range (OR = 0.91, [0.86–0.96]) (6) were associated with a higher odds of poor functional outcome, Table 2. Of note, only the association between SBP reduction and functional outcome differed according to the specific reperfusion status [i.e., mTICI 2B vs. mTICI 3, (13) Table 2]. SBP reduction was inversely associated with poor functional outcome in patients with mTICI 3 but not in mTICI 2B patients (13). The reperfusion status did not modify the association between maximum SBP and SBP range with functional outcome.

Only one study without post-EVT BP targets assessed the association between steady BP parameters and functional outcome. SBP mean (OR = 0.86 [0.79–0.93]) was the only steady BP parameter associated with poor outcome in this study (6). Interestingly, the latter association was consistent regardless of the specific reperfusion status (TICI 2B or TICI 3, Table 2).

### Relationship Between BP After Successful EVT and sICH

As depicted in Supplementary Tables 3, 4, there was a significant heterogeneity regarding sICH definition among the included studies. Overall, only one study specifically assessed the association between steady and dynamic BP parameters and sICH as a primary outcome (Table 1) (11).

#### Studies With Specific Post-EVT BP Targets

Among the studies with strict BP targets after successful EVT, only one dynamic BP parameter was associated with higher odds of sICH: time rate of SBP (OR = 1.71, [1.01–2.9]) (11). Of note, no steady BP parameters were associated with sICH in studies with BP control after successful EVT. Interestingly, sICH occurred in 3, 8, 5% in the intensive BP control group (SBP < 140 mmHg), moderate BP control group (SBP < 160 mmHg) and guideline-recommended group (SBP < 180 mmHg) and there was no significant association between these BP goals and the odds of sICH (30).

#### Studies Without Specific Post-EVT BP Targets

In contrast, more dynamic BP parameters were associated with sICH in studies without specific BP targets after successful EVT (Table 3). For dynamic BP parameters, SBP range (OR = 1.19, [1.08–1.30]) (6) and maximum SBP (OR = 1.23, [1.20–1.30]) (6) were associated with higher odds of sICH, regardless of the reperfusion status (TICI 2B vs. TICI 3). Interestingly, SBP SD (OR = 1.06, [1.01–1.13]) was associated with higher odds of sICH but this association differed according to the reperfusion status (significant in TICI 2B but not in TICI 3 patients, Table 3) (6). However, the analysis according to the specific reperfusion status was exploratory and only concerned a small number of patients (only 23 patients had sICH in the TICI 3 subgroup), limiting the interpretation of this result. Maximum and minimum DBP (OR = 1.19, [1.12–1.26] and OR = 1.33, [1.18–1.5], respectively) were also associated with higher odds of sICH, without any subgroup analysis available according to the reperfusion status (6).

**Table 3:** Blood pressure parameters associated with sICH in multivariate analysis, in the overall population and according to the specific reperfusion status.

**BP parameters**	**Results (overall)**	**Results according to the reperfusion status**
**Studies without specific post-EVT BP targets**		
***Dynamic BP parameters (BP variability)***		
**TR of SBP** (per 0.1 mmHg/min increase) (11)	OR = 1.71, 95%CI: 1.013–2.886, *p* = 0.045 had higher odds for sICH	
***Steady BP parameters***		
None		
**Studies without specific post-EVT BP targets**		
***Dynamic BP parameters (BP variability)***		
**SBP SD** **(****6****)**	OR = 1.06, (1.01–1.13), *p* = 0.018	***Patients with TICI3:*** No significant results ***Patients with TICI2B:*** OR = 1.09 (1.06–1.13), *p* = 0.010
**SBP range** **(****6****)**	OR = 1.19, (1.08–1.30), *p* < 0.001	***Patients with TICI3:*** OR = 1.27 (1.11–1.46), *p* = 0.001 ***Patients with TICI2B:*** OR = 1.13 (1–1.28), *p* = 0.044
**Maximum SBP** **(****6****)**	OR =1.23, (1.2–1.3), *p* < 0.001	***Patients with TICI3:*** OR = 1.27 (1.11-1.47), *p* = 0.001 ***Patients with TICI2B:*** OR = 1.22 (1.08–1.38), *p* = 0.002
**Maximum DBP** **(****6****)**	OR = 1.19, (1.12–1.26), *p* = 0.005	
**Minimum DBP** **(****6****)**	OR = 1.33, (1.18–1.5), *p* = 0.014	
***Steady BP parameters***		
**Mean SBP** **(****6****)**	OR = 1.29, (1.19–1.5), *p* = 0.003	***Patients with TICI3:*** No significant result ***Patients with TICI2B:*** OR = 1.32 (1.08–1.61), *p* = 0.007
**Mean DBP** **(****6****)**	OR = 1.38, (1.23–1.55), *p* = 0.001	

*BP, blood pressure; DBP, diastolic blood pressure; EVT, endovascular therapy; SBP, systolic blood pressure; SD, standard deviation; TICI, Thromboysis In Cerebral Infarction; TR, time rate*.

As for steady BP parameters, mean SBP, and mean DBP were associated with higher odds of sICH (6). Of note, the association between mean SBP and sICH differed according to the reperfusion status (significant in TICI 2B but not in TICI 3 patients, Table 3).

## Discussion

The present analysis suggests a potential varying effect of dynamic and steady BP parameters on functional outcome and sICH, respectively. Overall, SBP appeared to be the main parameter used. This systematic review highlights the significant heterogeneity in BP monitoring management, BP targets, and sICH definitions used after successful EVT across centers.

As previously described during EVT (5, 31), steady and dynamic BP parameters may also have a varying effect on functional outcome and sICH after successful EVT. No dynamic BP parameters were associated with functional outcome in case of BP control. This finding is of importance as BP control could prevent any abrupt BP variation responsible for infarct growth in case of successful but not complete reperfusion (i.e., mTICI 2B). This point has been recently illustrated by Anadani et al., in which SBP reduction after successful EVT was associated with an increased likelihood of good outcomes in patients with complete reperfusion (mTICI 3) but not in patients with successful but still incomplete reperfusion (mTICI 2B) (13). This hypothesis is consistent with previous data suggesting an infarct growth within days after treatment in case of successful recanalization but incomplete reperfusion (32, 33). Altogether, these data support the need to consider the exact reperfusion status after successful EVT for BP management. Indeed, mTICI 2B patients could be a population at risk and more prone to infarct growth in case of BP variability. Of note, three studies evaluated the reperfusion status with the mTICI score (6, 13, 30), but the other two studies assessed the reperfusion with the TICI score (11, 16). This difference could have had an impact on the grading of TICI 2 patients (some mTICI 2B, successfully reperfused patients being classified as TICI 2A in case of >50% but less than two-thirds) and could have influenced the studied associations.

In studies without strict BP protocols after EVT, several dynamic BP parameters were associated with increased odds of sICH. In contrast, only one study with a strict BP protocol displayed one BP parameter associated with sICH (Time rate of SBP: OR = 1.71, [1.01–2.9]) out of several BP variability parameters (11). On the contrary, no steady BP parameters were associated with sICH in case of BP targets after EVT. As stated by the authors, the TR of BP variation is a marker of beat-to-beat changes in BP and could therefore better reflect the speed of BP fluctuation and the change of consecutive BP data in time order, compared to traditional BP variability parameters (11). From a pathophysiological view, abrupt and sudden BP variability (including hypertension), taken into account with the TR of BP variation, could lead to blood-brain barrier breakdown and sICH (11, 34). These results seem crucial, as current recommended management (2) and future randomized controlled studies regarding BP after EVT only focus on fixed BP thresholds with no regards of BP variability. The present systematic review seems to underline how dynamic BP parameters are hidden factors that might have a strong negative impact on functional outcome and sICH occurrence. Further studies are needed to confirm the relevance of BP variability control on functional outcome and sICH respectively, so that the notion of BP fluctuation is considered in the upcoming guidelines.

Overall, two studies with strict BP control after EVT (SBP < 140 mmHg vs. SBP < 180 mmHg and SBP < 160 mmHg) described increased odds of favorable functional outcome but no such associations for sICH (16, 30). However, it remains difficult to explain with these evidences why this trend of favorable outcome (supposedly due to BP control) is not driven by a decreased rate of sICH. First, this result could be partly explained by the many different definitions of sICH among the included studies (Supplementary Table 4). Second, and as mentioned above, BP targets only concerned steady BP parameters (i.e., BP thresholds) and not BP variability control. As stated above, we found a differentiated effect of dynamic over steady BP parameters regarding sICH rates after EVT that could, partly, explain this finding. Third, this result must be interpreted with caution given from the fact that recent studies described an association between lower BP and lower odds of sICH (9, 21, 22). These studies were not included in the present work given their design (inclusion of patients with and without reperfusion, only subgroup analysis in reperfused patients). Finally, BP targets after EVT often result in greater use of anti-hypertensive treatments, some of which may have some neuroprotective properties independent of their BP lowering properties (35). Unfortunately, the exact type of antihypertensive treatment was not consistently reported. Morevover, intravenous thrombolysis use before EVT was not taken into account in these analyses. This point is worth mentioning, as IVT use is known to be associated with sICH occurrence and could have played a strong role according to different BP levels. Secondly, given the narrative nature of this review and possibly overlapping data in the included studies, further analysis could not be performed to test this association. Future studies are thus needed to assess the interaction in the relationship between BP parameters and sICH rates with IVT use before EVT.

In this systematic review, maximum DBP, minimum DBP, and mean DBP were strongly associated with sICH, but not with functional outcome. These associations are of great interest, as DBP is a rather rarely used BP parameter in AIS current practice. A recent study recently highlighted the impact of DBP on the cortical cerebral blood flow (36). As other low-resistance organs, the cerebral diastolic flow is crucial for the perfusion of the microvasculature (37). Recently, we also found an impact of DBP variability during EVT on functional outcome (31). Applied to the current study, it seems that maximum DBP and mean DBP were even more associated with sICH. As sICH are associated with microvasculature lesions in preclinical models, higher DBP levels after successful reperfusion could have had a crucial impact on small cerebral arteries and led to endothelial lesions, hence sICH. Clearly, this result deserves to be further explored through preclinical models of AIS and in larger populations in clinical research, to assess whether the impact of DBP is as relevant as that of SBP in the acute phase.

An individualized BP management after EVT, tailored to the patient's baseline characteristics could represent an alternative. To this end, Anadani et al. proposed a sub-analysis based on several key prognostic variables and found a possible varying effect of BP parameters among these characteristics (13). This tailored management is attracting growing attention, as shown by this recent study in which fixed BP thresholds were compared with autoregulation-oriented BP thresholds after EVT (38). By using Near-infrared spectroscopy-derived tissue oxygenation as a cerebral blood flow surrogate in response to mean arterial pressure (MAP) changes, an autoregulatory index was calculated to assess the BP ranges with autoregulation preservation (38). The percent time with MAP above the upper limit of autoregulation was associated with worse functional outcomes at 3 months and more hemorrhagic transformations (38). MAP was neither considered as a hemodynamic BP parameter, nor for BP targets in the present systematic review. However, MAP is relevant because of its indirect implication in cerebral autoregulation. This management deserves to be tested on larger cohorts before its generalization in everyday practice.

There are still uncertainties whether BP has a direct negative impact on functional outcome (or sICH), or is an important, but still indirect, marker of worse functional outcomes. The acute hypertension response is frequent in the setting of AIS and several factors, also known to be associated with worse functional outcomes, may trigger this response. Unfortunately, observational studies cannot fully resolve this issue, despite prospective collected data, large populations and elaborated multivariate analysis. To fully understand and resolve this issue, quality RCT are urgently needed. Several on-going RCT assessing the BP impact after successful EVT are proposing different designs. The *Blood Pressure After Endovascular Stroke Therapy-II* (BEST-II, NCT04116112) is a phase 2 study with the purpose to assess the safety of lowering BP after successful EVT (mTICI ≥ 2B). Three SBP targets ( ≤ 180 mmHg, < 160 mmHg, < 140 mmHg) will be assigned for 24 h post-EVT, primary outcomes being the final infarct volume at 36 h and the 3-month utility-weighted mRS. *Blood Pressure Target in Acute Stroke to Reduce hemorrhage After Endovascular Therapy* (BP-TARGET, NCT03160677) is a phase 2 RCT comparing two strategies after successful reperfusion: standard management (SBP < 185 mmHg) vs. intensive BP lowering with an SBP < 130 mmHg for 24 h (39). With the enrollment of 320 patients now completed, results regarding the rate of intracranial hemorrhagic complications are expected very soon and could hopefully clarify BP management after EVT. Finally, the *Outcome in Patients Treated With Intraarterial Thrombectomy—Optimal Blood Pressure Control* (OPTIMAL-BP, NCT04205305) will assess the impact of two BP targets (<140 vs. 180 mmHg) after successful reperfusion on the mRS, sICH and mortality. However, despite the expected results of these RCTs, the management of patients without successful reperfusion, as well as the management according to the precise reperfusion status or the impact of BP variability will not be considered. Design of future RCT could thus address these issues. One study could be based on autoregulation-oriented BP thresholds after EVT, using Near Infrared Spectroscopy as proposed by Petersen et al. (38). Another design could consist in defining a tighter BP target interval to respect after EVT to avoid any BP variability, as currently evaluated during EVT by the DETERMINE (*Effect of Individualized vs. Standard Blood Pressure Management during Mechanical Thrombectomy for Anterior Ischemic Stroke*) trial (NCT04352296).

The results herein must be cautiously interpreted given the wide heterogeneity between the included studies, their observational design and inherent shortcomings. Given the significant heterogeneity among the different hemodynamic parameters used, no meta-analysis of association was performed. Although a recent meta-analysis concerning BP management before, during and after AIS was recently published (26), this very analysis appropriately excluded overlapping data [i.e., studies involving the same population, which was the case in the present analysis for three studies from the same author (6, 13, 30)] and did not focus on successfully reperfused patients only. The present analysis therefore provides updated additional data and seems to point toward similar results, regarding the impact of BP values post-EVT and the need of quality RCT regarding this issue. For future directions, homogenization of practices regarding BP monitoring and management seem more than necessary, as it has been recently proposed (8).

## Conclusion

The present systematic review highlights the significant heterogeneity in BP monitoring management, BP targets, and sICH definitions used after successful EVT across centers. Given the absence of data regarding SBP time course reduction or the lack of studies evaluating the association between BP management, sICH occurrence and IVT use, no decisive conclusion can be made from this systematic review. However, identifying these limitations seem crucial, as it will allow further investigations in this important field. Upcoming RCT are urgently needed to clarify the medical management after EVT.

## Disclosure

MM declares consulting activities for Boerhinger Ingelheim, Acticor Biotech, Amgen, Air Liquide and lectures fees for Boerhinger Ingelheim, Acticor Biotech, Amgen and Medtronic. BL declares a research grant from Stryker, Penumbra, and Microvention.

## Author Contributions

BM, FD, and MM: study concept and design. BM and FD: acquisition of data. BM, FD, JL, SE, SH, J-PD, BL, MM, RB, and MP: analysis and interpretation of data. BM, FD, and MM: drafting of the manuscript. BM, FD, JL, SE, SH, J-PD, BL, MM, RB, and MP: critical revision of the manuscript for important intellectual content. JL: statistical analysis. All authors read and approved the final manuscript.

## Conflict of Interest

The authors declare that the research was conducted in the absence of any commercial or financial relationships that could be construed as a potential conflict of interest.

## Abbreviations

EVT: endovascular therapy
BP: blood pressure
SBP: systolic blood pressure
DBP: diastolic blood pressure
NIHSS: National Institutes of Health Stroke Scale
mTICI: modified Thrombolysis In Cerebral Ischemia
RCT: randomized controlled trial.
