# Supplementary material for: Impact of Blood Pressure After Successful Endovascular Therapy for Anterior Acute Ischemic Stroke: A Systematic Review
Source: Front Neurol. 2020 Oct 29;11:573382. doi: 10.3389/fneur.2020.573382 (PMC7659685; doi:10.3389/fneur.2020.573382)
Supplement: Supplementary file 1 [file Table_1.DOCX]

**SUPPLEMENTAL MATERIAL:**

1. **Supplemental Methods:**
   1. **Database research:**

For articles published after 2012 but with inclusion period before 2012, we manually checked the devices used and acknowledged the studies that met the previous criteria and also included studies whose inclusion period encompassed more years after 2012. The following words were used: “acute stroke(s)”, anoxia ischemia, cerebral”, “anoxia, brain”, “acute cerebrovascular accident(s)”, “anterior cerebral circulation infarction”, “thrombectomy”, “aspiration, mechanical”, “disruption, mechanical clot”, “endovascular procedure(s)”, “endovascular technique”, “blood pressure”, “blood pressure monitor”, “low blood pressure”, “high blood pressure”, Supplemental table I. We also performed a systematic search on Scopus database with the following words: (ALL (stroke  OR  ischemic  AND stroke  OR  acute  AND ischemic  AND stroke)  AND  ALL (endovascular  AND therapy  OR  mechanical  AND thrombectomy)  AND  ALL (blood  AND pressure  OR  hemodynamic  OR  blood  AND pressure  AND control  OR  systolic  OR  diastolic)). We restricted the study to human articles. Secondly, we cross-referenced the selected articles using Google Scholar to assess the eligibility of the articles that cited the selected articles. Finally, we assessed the eligibility of every reference in the selected papers. Articles were first selected on the base of their title and abstract, and then the whole manuscript was analyzed for articles that passed the selection with the abstract. Disagreements regarding the inclusion of an article were settled between the 2 authors (BM, FD).

The following data were extracted: name of the journal, year of publication, 1^st^ author’s name, retrospective or prospective study, mono or multicentric, inclusion period, number of patients, proportion of patients with medical history of hypertension, the median NIHSS, stroke localization, endovascular therapy (EVT) devices used, intravenous thrombolysis use, recanalization rates, sedation mode (general anesthesia or conscious sedation), BP modality assessment (invasive or non-invasive, intervals between every measure, number of measures), BP targets after EVT and measures (systolic blood pressure -SBP-, diastolic blood pressure -DBP- or mean arterial pressure –MAP- steady or dynamic parameters), hemodynamic treatment used after EVT (vasopressors or antihypertensive drugs), 3-month mRS according to post-EVT BP, definition of symptomatic intracranial hemorrhage (sICH), timeframe of post-EVT controlled brain imaging, sICH according to post-EVT BP.

**Supplemental Table I.** Search strategy developed for MEDLINE using MeSH terms

| **Search** | **Query** | **Items found** |
| --- | --- | --- |
| **#1** | Search **(((((((((((acute stroke[All Fields]) OR acute strokes[All Fields]) OR anoxia ischemia, cerebral[All Fields]) OR anoxia, brain[All Fields]) OR acute cerebrovascular accident[All Fields]) OR acute cerebrovascular accidents[All Fields]) OR anterior cerebral circulation infarction[All Fields]))** | 371382 |
| **#2** | Search **(((((((((((thrombectomies[All Fields]) OR thrombectomy[All Fields]) OR aspiration, mechanical[All Fields]) OR aspirations, mechanical[All Fields]) OR mechanical aspiration[All Fields]) OR mechanical aspirations[All Fields]) OR disruption, mechanical clot[All Fields]) OR endovascular procedure[All Fields]) OR endovascular procedures[All Fields]) OR endovascular technique[All Fields])))** | 171332 |
| **#3** | Search **((((blood pressure[All Fields]) OR blood pressure monitor[All Fields]) OR low blood pressure[All Fields]) OR high blood pressure[All Fields]))** | 978035 |
| **#1 AND #2 AND #3** | Search **((((((((((((((acute stroke[All Fields]) OR acute strokes[All Fields]) OR anoxia ischemia, cerebral[All Fields]) OR anoxia, brain[All Fields]) OR acute cerebrovascular accident[All Fields]) OR acute cerebrovascular accidents[All Fields]) OR anterior cerebral circulation infarction[All Fields])))) AND ((((((((((((thrombectomies[All Fields]) OR thrombectomy[All Fields]) OR aspiration, mechanical[All Fields]) OR aspirations, mechanical[All Fields]) OR mechanical aspiration[All Fields]) OR mechanical aspirations[All Fields]) OR disruption, mechanical clot[All Fields]) OR endovascular procedure[All Fields]) OR endovascular procedures[All Fields]) OR endovascular technique[All Fields]))))) AND (((((blood pressure[All Fields]) OR blood pressure monitor[All Fields]) OR low blood pressure[All Fields]) OR high blood pressure[All Fields])))** | 1639 |
| **#4** | Search **("2012/01/01"[Date - Entrez] : "2020/03/05"[Date - Entrez])** | 9197535 |
| **#1 AND #2 AND #3 AND #4** | Search **((((((((((((((((acute stroke[All Fields]) OR acute strokes[All Fields]) OR anoxia ischemia, cerebral[All Fields]) OR anoxia, brain[All Fields]) OR acute cerebrovascular accident[All Fields]) OR acute cerebrovascular accidents[All Fields]) OR anterior cerebral circulation infarction[All Fields])))) AND ((((((((((((thrombectomies[All Fields]) OR thrombectomy[All Fields]) OR aspiration, mechanical[All Fields]) OR aspirations, mechanical[All Fields]) OR mechanical aspiration[All Fields]) OR mechanical aspirations[All Fields]) OR disruption, mechanical clot[All Fields]) OR endovascular procedure[All Fields]) OR endovascular procedures[All Fields]) OR endovascular technique[All Fields]))))) AND (((((blood pressure[All Fields]) OR blood pressure monitor[All Fields]) OR low blood pressure[All Fields]) OR high blood pressure[All Fields]))))) AND ("2012/01/01"[Date - Entrez] : "2020/03/05"[Date - Entrez])** | **884** |

The search on Scopus database was performed on May 3^rd^, 2020 and yielded to 730 articles, 623 being published after 2012 and eligible for the present systematic review.

1. **Supplemental Results:**

**Supplemental Table II. Relationships between post-EVT blood pressure measures and main outcomes in studies with and without successful reperfusion**

| **Author, Year** | **Citation Nb^*^** | **Nb of patients** | **Functional outcome** | **sICH definition** | **Timeframe for brain imaging** | **sICH results** | **Inclusion in the systematic review or reasons for exclusion** |
| --- | --- | --- | --- | --- | --- | --- | --- |
| **Studies with specific post-EVT BP targets** | | | | | | |  |
| Kim et al,^1^ 2019 | 8 | 211 | Not mentioned | ***Hemorrhagic transformation (HTF)*** classified into 4 categories (HI-1, HI-2, PH-1, PH-2)  ***sICH:*** PH-2 combined with a neurologic deterioration of $\geq$4 points on the NIHSS from baseline | Median 1 day (IQR: 1-1) | HTF: N=88 (41.7%), HI-1: 11.4%, HI-2: 13.3%, PH-1: 6.1%, PH-2: 10.9%, sICH: N=20 (9.5%)  **Univariate analysis:** *sICH vs no sICH*  **Mean SBP:** 141.2±19.2 *vs* 130.0±18.9, **p=0.021**  **Maximum SBP:** 167.2±24.0 *vs* 155.0±24.2 mmHg, **p=0.033**  **SBP max-min**: 58.7±27.3 *vs* 46.4±21.1 mmHg, **p=0.017**  **DBP max-min:** 36.7±15.9 *vs* 29.3±11.8 mmHg, **p=0.011**  BP variability:  **SBP SD**: 15.2±7.1 *vs* 12.3±4.9 mmHg, **p=0.019**  **CV DBP (%):** 12.7±5.2 *vs* 10.3±4.0 mmHg, **p=0.014**  **TR of SBP:** 0.32±0.23 *vs* 0.20±0.08 mmHg/min, **p=0.025**  **TR of DBP:** 0.18±0.08 vs 0.13±0.06 mmHg/min, **p=0.028**  No significative results for: SBP minimum, SBP CV, SBP SV, DBP mean, DBP maximum, DBP minimum, DBP SD, DBP SV  **Multivariate analysis:**  **TR of SBP** (per 0.1 mmHg/min increase):  **OR= 1.71, 95%CI: 1.013–2.886, p=0.045** had higher odds for sICH  No significative results for the other parameters | Included |
| Zhang et al,^2^ 2019 | 0 | 72 | **Univariable analysis:**  *BPV and 3-month mRS: mRS 3-6 vs 0-2*  **Maximum SBP:** 163.5±15.6 *vs* 154.3±16.8, **p=0.02**  **Systolic CV:** 11.0%±1.8% vs. 8.8%± 2.0%, **p<0.001**  **SV:** 14.6±2.0 *vs* 11. 4±2.3, **p<0.001**  **SD:** 13.8±3.9 *vs* 10.5±2.4, **p<0.001**  No significative results for MAP, mean SBP, minimum SBP, DBP variability  **Subgroup analysis:**  **- Patients with successful reperfusion:** *mRS 0-2 vs 3-6*  **Maximum SBP:** 153.4±15.9 vs 165.4±16.0, **p=0.006**  **Systolic SV:** 11.4±2.3 *vs* 14.4±2.0, **p<0.001**  **Systolic SD:** 10.0±2.4 *vs* 14.0±4.3, **p<0.001**  **Systolic CV:** 8.8%±2.0 *vs* 10.8%±1.7, **p<0.001**  No significant results for the other parameters  **- Patients without successful reperfusion:** **no** significant results for all parameters tested  **Predictive factors of poor outcome:**  **Univariable logistic regression:**  **Systolic SD post-EVT:** OR=1.531 (1.203-1.948), **p=0.001**  **Systolic SV post-EVT:** OR=2.046 (1.444-2.898), **p<0.001**  **Multivariable logistic regression:**  **Systolic SV post-EVT:** OR=4.273 (1.030-17.727), **p=0.045** | ICH defined by HI-1, HI-2, PH-1, PH-2  No clear definition of sICH | In the first 24h after EVT | HI-1: N=5 (6.9%); HI-2: N=5 (6.9%); PH-1: N=2 (2.8%); PH-2: N=1 (1.4%)  No association between post-EVT BP and ICH or sICH. | Excluded  Reason: main analysis concerned either reperfused and non-reperfused patients. |
| Chang et al,^3^ 2019 | 0 | 90 | **Univariable analysis:**  SBP$\leq$130 mmHg *vs* SBP>130 mmHg  For mRS$\leq$2: N=32 (66.7%) *vs* 17 (40.5%), **p=0.02**  **Multivariable analysis (multivariable ordinal logistic regression):**  **Mean SBP>130 mmHg:** **OR=2.66 (1.11–6.41)**  No significant results for the other parameters (24h mean SBP, percent reduction >15%) | HTF: HI-1, HI-2, PH-1, PH-2.  No clear definition of sICH | Not mentioned | HI: N=7 (7.8%), PH: N=16 (17.8%)  **24h mean SBP<=130 mmHg *vs* SBP>130 mmHg:** p=0.27  HI: 5 (10.4%) *vs* 2 (4.8)  PH: 6 (12.5%) *vs* 10 (23.8%) | Included |
| Anadani et al,^4^  2020 | 0 | 1019 | **Univariable analysis:**  Good functional outcome (mRS: 0-2):  SBP<140 mmHg: 52%  SBP<160 mmHg: 52%  SBP<180 mmHg: 44%  **Inverse probability of treatment weights (IPTW)-adjusted multivariate analysis for SBP<140 mmHg:** associated with higher odds of good functional outcome compared to SBP<180 mmHg: OR=1.53 (1.07-2.19)  For patients with pre-treatment SBP$\geq$140 mmHg: Intensive (SBP<140 mmHg) and moderate (SBP<160 mmHg) BP targets had higher odds of good functional outcome than guideline-recommended BP targets: OR=1.75 (1.07-2.85) and OR=2.30 (1.17-4.52), respectively. | sICH defined as any intracranial hemorrhage within 72 hours associated with $\geq$4 points increase in the NIHSS or death following EVT. | Within 72h | sICH in intensive BP target: 3%  sICH in moderate BP target: 8%  sICH in guideline-recommended target: 5%  **On IPTW-adjusted multivariable analysis:** no significant association between BP targets and sICH  For patients with pre-treatment SBP$\geq$140 mmHg:  Intensive BP group had lower odds of sICH compared to guideline-recommended group: OR=0.38 (0.16-0.91) | Included |
| Anadani et al,^5^ 2019 | 4 | 298 | **Univariate analysis:** *mRS 0-2 vs 3-6*  **SBP average (mean):** 121 (11.5) *vs* 125.5 (12.5), **p<0.001**  **SBP maximum:** 147.9 (20.5) *vs* 152.5 (18.3), **p<0.05**  No significant results for SBP minimum, SBP range, SBP SD and SBP CV.  **Multivariate analysis:**  Multivariate analysis between average SBP and favorable functional outcome: **OR=0.97 (0.940–0.998), p=0.026**  Multivariate analysis between maximum SBP and favorable functional outcome: OR=1 (0.98-1.01), p=0.634  Multivariable logistic regression analysis between favorable functional outcome and average SBP in TICI 2B/3 patients:  **OR=0.98 (0.96-0.999), p=0.044**  **No correlation in patients without successful reperfusion** | HTF: HI-1, HI-2, PH-1, PH-2  Only PH-2 included for multivariate analysis.  No clear definition of sICH | Not mentioned but ECASS II criteria | HTF: N=109 (36.6%), PH-2: N=17 (5.7%)  Multivariable analysis between average and maximum SBP and PH-2: no significant association. No analysis according to the reperfusion status.  No significant results for the association between HTF or PH-2 with average SBP<120 vs $\geq$120 mmHg in univariate analysis. | Included |
| McCarthy et al,^6^ 2019 | 1 | 212 | **Univariate analysis:** *mRS 0-2 vs 3-6*  **Mean maximum SBP:**  Day 1: 153±21 *vs.* 163±26, **p=0.002**  Day 2: 145±16 *vs.* 152±21, **p=0.008**  Day 3: 142 ± 18 *vs.* 156 ± 20, **p<0.0001**  **Univariate analysis according to the recanalization status:**  Patients with successful reperfusion (*mRS 0-2 vs 3-6*):  **Mean maximum SBP:**  Day 1: 154±21 *vs.* 161±25, p=0.067  Day 2: 146 ± 16 *vs.* 151 ± 22, p=0.09  Day 3: 143±17 vs. 156±19, **p<0.0001**  **Sensitivity analysis:**  **TICI 2B/2C:** *mRS 0-2 vs 3-6*  **SBP peak:**  Day 2, 144±12 *vs.* 153±21, **p=0.03**  Day 3, 142±17 *vs.* 155±20, **p=0.006**  **TICI 3:**  **SBP peak:**  No significant result at Day 1 or 2.  Day 3: 143±17 *vs.* 156±17, **p=0.0003**  **Increase in daily maximum SBP:**  From day 2 to 3: −3.4±16 *vs.* 5.8±20, **p=0.01**)  **Multivariate analysis:**  **Day 1 maximum SBP:** OR=0.85, 0.73–0.98, **p=0.031**  **Day 2 maximum SBP:** OR=0.90, 0.84-0.95, **p=0.042**  **Day 3 maximum SBP:** OR=0.59, 0.47-0.74, **p<0.0001**  **Peak SBP change from day 2 to day 3:** OR= 0.97, 0.95-0.99, **p=0.006**  No significant result for Peak SBP change from day 1 to 2.  Youden index split BP parameters associated with functional independence:  **Day 3 SBP below 140 mmHg**: OR=4.3, 1.96-9.9, **p=0.004** | sICH: presence of hemorrhagic conversion or space occupying hematoma accompanied with a 24h NIHSS increase of at least 4 points | 24 hours | Asymptomatic hemorrhage and hemorrhagic conversion: N=52 (24.5%)  Symptomatic hemorrhage and hemorrhagic conversion: N=15 (7.1%)  **Multivariable analysis:** association with sICH  **Day 1 SBP peak:** (per incremental of 10 mmHg increases): OR=1.2, 95%CI 1.01–1.49, **p=0.048** | Excluded  Reason: main analysis concerned either reperfused and non-reperfused patients. |
| Goyal et al,^7^ 2018 | 21 | 88 | **Univariate analysis:** *mRS 0-2 (yes vs no)*  **Maximum SBP:** 160±19 *vs.* 179±23, **p=0.001**  **Minimum SBP:** 119±12 *vs*. 108±25, **p=0.008**  **Mean SBP:** 140±13 *vs.* 141±17 p=0.798  **Maximum DBP:** 88±22 *vs.* 96±22, p=0.157  **Minimum DBP:** 54±11 *vs.* 55±12, p=0.757  **Mean DBP:** 71±11 *vs.* 72±12, p=0.579  **Univariable logistic regression analysis depicting the associations of BP and mRS 0-2:**  **Minimum SBP post MT (per 10 mmHg increase):** OR=1.31 (1.00 to 1.72), **p=0.048**  **Maximum SBP post MT (per 10 mmHg increase):** OR=0.67 (0.51 to 0.87), **p=0.003**  **Multivariable logistic regression analysis depicting the associations between BP and mRS 0-2:**  **Minimum SBP post EVT:** OR=1.64 (1.04 to 2.60), **p=0.033**  **Maximum SBP post EVT:** OR=0.55 (0.39 to 0.79), **p=0.001** | PH-2 on CT or T2* MRI accounting for neurologic deterioration with an increase >4 points on the NIHSS | Within 36h from treatment | sICH: N=8 (9%)  **Univariate analysis:** *sICH yes vs no*:  Maximum SBP: 183±31 vs 173±22, p=0.279  Minimum SBP: 105±18 vs 111±22, p=0.430  Mean SBP: 139±18 vs 141±15, p=0.698  Maximum DBP: 97±25 vs 94±20, p=0.692  Minimum DBP: 51±9 vs 55±12, p=0.384  Mean DBP: 74±12 vs 72±12, p=0.710 | Excluded  Reason: main analysis only concerned non-recanalized patients. |
| Goyal et al,^8^ 2017 | 60 | 217 | **Univariate analysis:** *mRS 0-2 vs 3-6*  **Maximum SBP during 24h:** 163±20 *vs.* 179±23, **p<0.001**  **Maximum DBP during 24h:** 91±15 mmHg *vs.* 97±14, **p=0.008**  No other significant results for SBP, DBP or MAP  **Multivariable logistic regression models:** association of maximum SBP and DBP levels (per 10 mmHg increase) with mRS 0-2:  **Maximum SBP:** OR=0.70 (0.56–0.87), **p=0.001**  **Maximum DBP:** OR=0.83 (0.63–1.08), p=0.171  Multivariable associations of different post-EVT BP targets and mRS 0-2: p=0.328  **Permissive:** OR=1 (reference)  **Moderate:** OR=2.75 (0.73–10.35), p=0.136  **Intensive:** OR=1.26 (0.20–8.12), p=0.809 | PH-2 on CT or T2* MRI accounting for an increase >4 points on the NIHSS | Within 36h from treatment | Asymptomatic ICH: N=64 (30%)  sICH: N=14 (6.5%)  **Univariate analysis:** *sICH vs. no sICH*  **Maximum SBP during 24h:** 173±25 vs 171±22, p=0.793  **Maximum DBP during 24H:** 100±18 vs 94±15, p=0.129  sICH according to the subgroup of BP target:  Intensive: N=1 (10%), Moderate: N=2 (5.6%), Permissive: N=5 (5.3%), p=0.831 | Excluded  Reason: main analysis concerned either reperfused and non-reperfused patients. |
| Chu et al,^9^ 2019 | 0 | 224 | **Multivariate analysis of the association between SBP and DBP with functional independence at 3 months:**  SBP mean 1-6h: OR=0.962 (0.933-0.990), **p=0.009**  SBP mean 7-12h: OR=0.973 (0.945-1.001), p=0.057  SBP mean 13-18h: OR=0.975 (0.946-1.005), p=0.101  SBP mean 19-24h: OR=0.975 (0.946-1.006), p=0.116  SBP maximum 1-6h: OR=0.961 (0.940-0.982), **p<0.001**  SBP maximum 7-12h: OR=0.970 (0.947-0.993), **p=0.011**  SBP maximum 13-18h: OR=0.969 (0.943-0.996), **p=0.022**  SBP maximum 19-24h: OR=0.969 (0.943-0.996), **p=0.026**  SBP SD 1-6h: OR=0.870 (0.809-0.936), **p<0.001**  SBP SD 7-12h: OR=0.895 (0.819-0.977), **p=0.013**  SBP SD 13-18h: OR=0.896 (0.807-0.996), **p=0.042**  SBP SD 19-24h: OR=0.852 (0.762-0.951), **p=0.004**  DBP mean 1-6h: OR=0.943 (0.908-0.980), **p=0.002**  DBP mean 7-12h: OR=0.952 (0.914-0.991), **p=0.017**  DBP mean 13-18h: OR=0.960 (0.919-1.003), p=0.068  DBP mean 19-24h: OR=0.961 (0.924-1.001), p=0.054  DBP maximum 1-6h: OR=0.957 (0.932-0.982), **p<0.001**  DBP maximum 7-12h: OR=0.970 (0.941-0.999), **p=0.045**  DBP maximum 13-18h: OR=0.974 (0.944-1.005), p=0.099  DBP maximum 19-24h: OR=0.978 (0.951-1.006), p=0.123  DBP SD 1-6h: OR=0.900 (0.830-0.975), **p=0.010**  DBP SD 7-12h: OR=0.948 (0.856-1.050), p=0.303  DBP SD 13-18h: OR=0.990 (0.898-1.090), p=0.834  DBP SD 19-24h: OR=0.962 (0.868-1.067), p=0.464 | Any intracranial hemorrhage that was accompanied by a $\geq$4 points increase in NIHSS scores or that led to death within 24h | Within 24h | sICH: N=13 (5.8%)  No analysis between sICH and BP post-EVT | Excluded  Reason: main analysis concerned either reperfused and non-reperfused patients. |
| **Studies without specific post-EVT BP targets** | | | | | | |  |
| Cho et al,^10^ 2019 | 4 | 378 | **Univariate analysis:** *mRS 0-2 vs 3-6*  **SBP mean:** 124.2±13.0 *vs* 129.9±14.6, **p<0.001**  **SBP max:** 154.5±31.5 *vs* 160.4±20.7, **p=0.044**  **SBP SV:** 9.8±4.1 *vs* 12.2±4.9, **p<0.001**  **DBP mean:** 68.6±7.6 *vs* 71.9±9.0, **p<0.001**  **DBP SD:** 8.2±3.3 *vs* 9.0±2.9, **p=0.022**  **DBP max:** 87.7±12.6 *vs* 92.3±12.9, **p<0.001**  **DBP SV:** 6.3±2.1 *vs* 7.8±3.1, **p<0.001**  **Multivariate analysis:** logistic regression model for the association between continuous SBP levels and mRS 0-2:  ***All patients:***  **SBP Mean:** OR=0.82 (0.69–0.97), **p=0.02**  **SBP SV:** OR=0.38 (0.19–0.76), **p=0.01**  **DBP mean:** OR=0.60 (0.44-0.82), **p=0.001**  **DBP SV:** OR=0.20 (0.07-0.59), **p=0.004**  No significant results for the other parameters.  ***Patients with successful reperfusion:***  **SBP mean:** OR=0.78 (0.65–0.94), **p=0.01**  **SBP SV:** OR=0.27 (0.12–0.62), **p=0.002**  **DBP mean:** OR=0.57 (0.41-0.80), **p=0.001**  **DBP SV:** OR=0.17 (0.05-0.59), **p=0.01**  No significant results for the other parameters.  ***Patients without successful reperfusion:***  No significant results for every SBP and DBP parameters. | PH-2 and an increase in NIHSS $\geq$4 points | 24 hours and day 5 | sICH=60 (16.9%)  No significant results in univariate or multivariate analysis between SBP or DBP parameters and sICH. | Excluded  Reason: main analysis concerned either reperfused and non-reperfused patients. |
| Chang et al,^11^ 2018 | 6 | 303 | **Univariate analysis:** *mRS adjusted^e^ good vs poor*  **24h SBP Mean:** 124.34±12.46 *vs.* 129.04±14.06, **p<0.01**  **24h SBP SD:** 12.26 (9.60–15.53) *vs.* 14.70 (11.79–19.27), **p<0.01**  **24h DBP SD:** 8.83 (7.07–10.75) *vs.* 10.85 (8.66–13.45), **p<0.01**  **24h SBP CV:** 0.10 (0.08–0.12) *vs.* 0.11 (0.09–0.14), **p<0.01**  **24h DBP CV:** 0.13 (0.10–0.16) *vs.* 0.15 (0.12–0.20), **p<0.01**  **24h SBP VIM:** 0.10 (0.08–0.12) *vs.* 0.12 (0.09–0.15), **p<0.01**  **24h DBP VIM:** 0.13 (0.10–0.15) *vs.* 0.16 (0.12–0.19), **p<0.01**  **48h SBP Mean:** 122.27±12.79 *vs.* 128.38±15.08, **p<0.01**  **48h SBP SD:** 10.37 (8.65–12.31) *vs.* 12.22 (10.01–15.77), **p<0.01**  **48h DBP SD:** 8.92 (6.81–10.81) *vs.* 9.63 (7.47–12.88), **p=0.01**  **48h SBP CV:** 0.08 (0.07–0.10) *vs.* 0.10 (0.07–0.12), **p<0.01**  **48h DBP CV:** 0.12 (0.10–0.16) *vs.* 0.14 (0.11–0.19), **p<0.01**  **48h SBP VIM:** 0.08 (0.07–0.10) *vs.* 0.10 (0.07–0.12), **p<0.01**  **48h DBP VIM:** 0.13 (0.10–0.15) *vs.* 0.13 (0.11–0.18), **p=0.02**  **Multivariate analysis:**  ***mRS*** $\boldsymbol{\leq}$***1:***  **48h SBP Mean:** OR=0.76 (0.59-0.97)  ***mRS*** $\boldsymbol{\leq}$***2:***  **24h DBP SD**: OR=0.66 (0.47–0.94)  **24h DBP CV:** OR=0.67 (0.47–0.96)  **24h DBP VIM:** OR=0.66 (0.47–0.94)  **48h SBP Mean:** OR=0.79 (0.63–0.99)  **48h SBP SD:** OR=0.68 (0.47–0.98)  ***mRS ordinal logistic regression:***  **24h SBP SD:** OR=1.5 (1.20–1.88)  **24h DBP SD:** OR=1.64 (1.31–2.05)  **24h SBP CV:** OR=1.47 (1.17–1.85)  **24h DBP CV:** OR=1.56 (1.24–1.97)  **24h SBP VIM:** OR=1.48 (1.18–1.86)  **24h DBP VIM:** OR=1.64 (1.31–2.05)  **48h SBP Mean:** OR=1.26 (1.07–1.48)  **48h SBP SD:** OR=1.55 (1.22–1.96)  **48h SBP CV:** OR=1.44 (1.14–1.82)  **48h SBP VIM:** OR=1.39 (1.10–1.76) | Not clearly mentioned | Not mentioned | sICH: N=17 (6.1%)  No analysis between sICH and BP post EVT | Excluded  Reason: main analysis concerned either reperfused and non-reperfused patients. |
| Mistry et al,^12^ 2019 | 3 | 485 | The peak SBP that best discriminated poor and favorable outcome was **158 mmHg** (Se=52%, Sp=68%, AUC=0.61, 95%CI: 0.56-0.66, **p<0.001**)  **With peak SBP>158 mmHg**:  Unadjusted OR=2.24 (1.52-3.29) Adjusted OR=1.29, (0.81–2.06), p=0.28 for having a bad outcome  **Analysis with SBP as continuous:**  unadjusted OR=1.02 (1.01–1.03), **p<0.01** adjusted OR=1.00 (0.99–1.01), p=0.79  **mRS (shift analysis) and SBP dichotomized:**  Unadjusted OR= 2.27 (1.62–3.19), **p<0.01** Adjusted OR=1.25 (0.87–1.81), p=0.22  No evidence of a differential effect of higher peak SBP on bad outcome by mTICI status | sICH: any intracranial hemorrhage associated with $\geq$4 points increase in NIHSS from baseline to 24h | Within 72 hours | ICH: N=117 (24%)  sICH: N=18 (4%)  No significant results between SBP peak (dichotomized or continuous analysis) and any ICH or sICH. | Excluded  Reason: main analysis concerned either reperfused and non-reperfused patients. |
| Mistry et al,^13^ 2020 | 1 | 443 | Three multivariate models tested for the association between mRS 3-6 and the risk of being in the highest compared with the lowest tertile of BPV:  **Results of Model 2:**  ***For SBP:***  **SD:** OR=1.85 (1.04-3.29), **p=0.035**  **rSD:** OR=2.93 (1.59–5.38), **p=0.001**  **CV:** OR=1.79 (1.02–3.15), **p=0.042**  **ARV:** OR=1.61 (0.91–2.85), **p=0.099**  **SV:** OR=1.84 (1.04–3.26), **p=0.037**  ***For DBP:***  No significant results with Model 2  Only **CV** for **model 3**, OR=2.34 (1.35–4.05), **p=0.002**  Stratification by the reperfusion status:  Patients with TICI 3:  OR for poor outcome, adjusted for SBP mean: OR=3.10; 95% CI, 1.54–6.23; **p=0.002**  No significant results in patients with TICI2B or TICI<2B | sICH: any ICH associated with $\geq$4 points increase in NIHSS from baseline to 24h | Within 72 hours | Not mentioned | Excluded  Reason: main analysis concerned either reperfused and non-reperfused patients.  Secondary analysis according to the reperfusion status (stratification). |
| Anadani et al,^14^ 2019 | 0 | 1361 | **rSBP and poor outcome:**  Model 1: OR=0.99 (0.98-0.99), **p=0.007**  Model 2: OR=0.97 (0.95-0.98), **p<0.001**  ***Subgroup analysis in patients with TICI 3:***  Model 1: OR=0.98 (0.97 to 0.99), **p=0.010**  Model 2: OR=0.98 (0.97 to 0.98), **p=0.014**  ***Subgroup analysis in patients with TICI 2B:***  Model 1: no significant results  Model 2: OR=0.99 (0.98 to 1.007)  SBPr included as a categorical variable (<1% (reference), 1%–10%, 11%–20%,>20%):  **1%–10% category:** OR=0.7; 95% CI 0.45 to 0.99  No significant results for >10% | Intraparenchymal, subarachnoid or intraventricular hemorrhage on post procedural CT associated with $\geq$4 NIHSS points during the first 72h after EVT | Within 72H | No significant association between rSBP and sICH | Included |
| Mistry et al,^15^ 2017 | 54 | 228 | **Adjusted OR from multivariable ordinal regression analysis, N=190:**  ***For SBP:***  **Maximum:** OR=1.02 (1.01–1.03), **p<0.004**  **Minimum:** OR=1.00 (0.99–1.02), p<0.1  **Average:** OR=1.02 (1.00–1.04), p=0.05  ***For DBP:***  No significant results for maximum, minimum and average  ***For MAP:***  No significant results for maximum, minimum and average  **Subgroup analysis according to the reperfusion status:**  ***Patients with successful reperfusion:***  Maximum SBP and worse outcome: aOR=1.02 (1.00-1.03), **p=0.01**; N=56 | Any intracranial hemorrhage within 48h after EVT on brain imaging with an increase $\geq$4 points on the NIHSS | Within 48 hours | **Adjusted OR from multivariable ordinal regression analysis of hemorrhagic complications** (from no ICH to asymptomatic ICH to sICH), N=228:  ***For SBP:***  **Maximum:** OR=1.02 [1.01–1.04], **p<0.002**  No significant results for minimum and average  For DBP: no significant results for maximum, minimum and average  For MAP: no significant results for maximum, minimum and average  **Subgroup analysis according to the reperfusion status:**  ***- Patients with successful reperfusion:***  **Maximum SBP** correlated with severity of ICH: aOR=1.02 (1.00-1.03), **p=0.05**, N=183)  **Minimum DBP** inversely correlated with ICH complications: aOR=0.96 (0.93-1.00), **p=0.04**  ***- Patients without successful reperfusion:***  **Maximum SBP**: aOR=1.05 (1.01-1.10), **p=0.01**  **Maximum MAP**: aOR=1.06 (1.01-1.11), **p=0.02** | Excluded  Reason: main analysis concerned either reperfused and non-reperfused patients. |
| Anadani et al,^16^ 2019 | 4 | 1245 | **Univariable analysis:** *mRS 3-6 vs 0-2*  **Mean SBP:** 131 (16) vs 127 (14), **p<0.001**  **Maximum SBP:** 164 (25) vs 157 (24), **p<0.001**  **Minimum SBP:** 101 (18) vs 101 (15), p<0.999  **SBP SD:** 14 (5) vs 13 (5); **p=0.018**  **SBP Range:** 62 (26) vs 56 (24), **p<0.001**  **Multivariate analysis:**  **Mean SBP:** OR=0.86 (0.79–0.93), **p<0.001**  **Maximum SBP:** OR=0.9 (0.85–0.95), **p<0.001**  **SBP SD:** OR=0.99 (0.96–1.02), p=0.43  **SBP range:** OR=0.91 (0.86–0.96), **p=0.003**  No significant results for DBP parameters  SBP 101-120, 141-160 and >160 mmHg:  **SBP 141-160** and **>160 mmHg** had **43%** and **66%** lower odds of achieving good outcome at 90 days in comparison to 121-140 mmHg  mRS 0-2 rates were the highest for **101-120 mmHg** (**55%**) and the lowest for **>160 mmHg**. | sICH: any intraparenchymal, subarachnoid, intraventricular hemorrhage on post-procedural CT associated with $\geq$4 points on the NIHSS | Not mentioned but ECASS criteria | **Univariable analysis:** *no sICH vs sICH*  **Mean SBP:** 129 (15) vs 136 (15), **p<0.001**  **Maximum SBP:** 160 (24) vs 175 (27), **p<0.001**  **SBP SD:** 14 (5) vs 16 (7), **p=0.029**  **SBP range:** 59 (25) vs 71 (29), **p=0.002**  **Multivariate analysis:**  **Mean SBP:** OR=1.29 (1.19–1.5), **p=0.003**  **Maximum SBP:** OR=1.23 (1.2–1.3), **p<0.001**  **SBP SD:** OR=1.06 (1.01–1.13), **p=0.018**  **SBP range:** OR=1.19 (1.08–1.30), **p<0.001**  ***SBP as a categorial variable:***  **mean SBP** between **101-120 mmHg**: lower odds of sICH than the reference group: OR=0.37, **p=0.006**  For patients with SBP 141-160 and >160 mmHg: non-significant higher odds of sICH  The rate of sICH was the highest for the **SBP>160 mmHg** group (11%) *vs* **<100 mmHg** group (0%) | Included |
| Cernik et al,^17^ 2019 | 6 | 690 | **Univariate analysis:** *mRS 0-2 vs 3-6*  **SBP mean (SD):** 133±15 *vs* 139±16, **p<0.001**  **DBP mean (SD):** 69±9 *vs* 70±10, p=0.094  **Maximum SBP mean (SD):** 161±22 *vs* 172±24, **p<0.001**  **Maximum DBP mean (SD):** 90±15 *vs* 93±15, **p=0.002**  Multivariable logistic regression model for good 3-month clinical outcome:  **Median DBP:** OR=0.977 (0.957 to 0.997), **p=0.024**  No significant results for the other parameters  ***Patients with successful reperfusion:***  **Median max SBP:** OR=0.990 (0.981 to 0.999), **p=0.038**  No significant results for the other parameters  ***Patients with TICI 3:***  **Median max DBP:** OR=0.984 (0.969 to 1.000), **p=0.046**  No significant results for the other parameters | Local remote parenchymal hematoma type 2 or subarachnoid hemorrhage associated with $\geq$4 points NIHSS or leading to death. | Within 24 hours | **Univariate analysis:**  Median SBP <140 mmHg *vs* median SBP $\geq$140 mmHg:  sICH: 19 (5.1%) *vs* 16 (5.1%), p=0.980  sICH *vs* no sICH:  No significant results for the other BP parameters  Maximal SBP (median, range): 175 (135-230) *vs* 165 (130-250), **p=0.029** | Excluded  Reason: main analysis concerned either reperfused and non-reperfused patients. |
| Matusevicius et al,^18^ 2020 | 0 | 3631 | ***Patients with successful reperfusion:*** SBP range of 100 to 119 mmHg had the highest percentage of functional independence (63%)  ***Patients without successful reperfusion:***  SBP range of 120 to 139 mmHg had the highest percentage of functional independence (31%)  **Multivariable logistic regression models**:  -SBP as a continuous independent variable  ***Patients with successful reperfusion:*** aOR=0.987 (0.980–0.995)  (Higher SBP values in the linear model was independently associated with less functional independence)  ***Patients without successful reperfusion:*** no association with mRS.  -DBP as a continuous variable:  ***Patients with successful reperfusion:***  OR=0.987 (0.975–0.998), (an increase in DBP was associated with less functional independence)  ***Patients without successful reperfusion***: no association with mRS  -SBP as interval categorical variable:  ***Patients with successful reperfusion:***  Compared with the reference interval of 100 to 119 mmHg:  **SBP intervals of 140 to 159:** aOR=0.65, (0.43–0.97)  **SBP≥160 mm Hg:** aOR=0.28, (0.14– 0.53) (association with less 3-month functional independence) | Local remote parenchymal hematoma type 2 or subarachnoid hemorrhage associated with $\geq$4 points NIHSS or leading to death. | Within 22-36H | **Multivariable logistic regression models:**  -SBP as a continuous independent variable:  ***Patients with successful reperfusion***: aOR=1.027, (1.007– 1.047), (higher SBP values in the linear model was independently associated with sICH)  ***Patients without successful reperfusion:*** aOR=1.040, (1.008–1.075), (higher SBP values were associated with more sICH)  - DBP as continuous variable:  ***Patients with successful reperfusion:***  OR=1.033, (1.001–1.066), (an increase in DBP was associated with more sICH)  ***Patients without successful reperfusion***: OR=1.072, (1.010–1.142), (association between higher DBP and sICH)  -SBP as interval categorical variable:  ***Patients with successful reperfusion:***  Compared with the reference interval of 100 to 119 mm Hg:  **SBP≥160 mmHg:** aOR=6.82, (1.53–38.09)  Compared with the reference interval of 120 to 139 mmHg,  **SBP ≥160 mmHg**: aOR=6.62, (1.07–51.05) | Excluded  Reason: main analysis concerned either reperfused and non-reperfused patients. |

**Abbreviations:** BP: blood pressure; BPV: BP variability; EVT: endovascular therapy; Nb: number; SBP: systolic blood pressure; DBP: diastolic blood pressure; MAP: mean arterial pressure; mRS: modified Rankin Scale; HTF: hemorrhagic transformation; ICH: intracranial hemorrhage; sICH: symptomatic intracranial hemorrhage

**^a^** SD means standard deviation of the mean

^b^ SV means successive variation (square root of the average of squared difference between successive BP measurements)

^c^ CV means coefficient of variation (SD/mean BP)

^d^ TR means time rate

^e^ Good outcome is defined as a 3-month mRS score of 0 to 1 for pretreatment NIHSS scores of ≤7, mRS score of 0–2 for pretreatment NIHSS scores of 8–14, and mRS scores of 0–3 for pretreatment NIHSS scores of >14.

*Google scholar searched performed on May 3^rd^ 2020

**Supplemental Table III. Relationships between post-EVT blood pressure measures and main outcomes in studies with successful reperfusion**

| **Author, Year** | **Citation Nb^*^** | **Nb of patients** | **Functional outcome** | **sICH definition** | **Timeframe for brain imaging** | **sICH results** |
| --- | --- | --- | --- | --- | --- | --- |
| **Studies with specific post-EVT BP targets** | | | | | | |
| Kim et al,^1^ 2019 | 8 | 211 | Not mentioned | ***Hemorrhagic transformation (HTF)*** classified into 4 categories (HI-1, HI-2, PH-1, PH-2)  ***sICH:*** PH-2 combined with a neurologic deterioration of $\geq$4 points on the NIHSS from baseline | Median 1 day (IQR: 1-1) | HTF: N=88 (41.7%), HI-1: 11.4%, HI-2: 13.3%, PH-1: 6.1%, PH-2: 10.9%, sICH: N=20 (9.5%)  **Univariate analysis:** *sICH vs no sICH*  **Mean SBP:** 141.2±19.2 *vs* 130.0±18.9, **p=0.021**  **Maximum SBP:** 167.2±24.0 *vs* 155.0±24.2 mmHg, **p=0.033**  **SBP max-min**: 58.7±27.3 *vs* 46.4±21.1 mmHg, **p=0.017**  **DBP max-min:** 36.7±15.9 *vs* 29.3±11.8 mmHg, **p=0.011**  BP variability:  **SBP SD**: 15.2±7.1 *vs* 12.3±4.9 mmHg, **p=0.019**  **CV DBP (%):** 12.7±5.2 *vs* 10.3±4.0 mmHg, **p=0.014**  **TR of SBP:** 0.32±0.23 *vs* 0.20±0.08 mmHg/min, **p=0.025**  **TR of DBP:** 0.18±0.08 vs 0.13±0.06 mmHg/min, **p=0.028**  No significative results for: SBP minimum, SBP CV, SBP SV, DBP mean, DBP maximum, DBP minimum, DBP SD, DBP SV  **Multivariate analysis:**  **TR of SBP** (per 0.1 mmHg/min increase):  **OR= 1.71, 95%CI: 1.013–2.886, p=0.045** had higher odds for sICH  No significative results for the other parameters |
| Chang et al,^3^ 2019 | 0 | 90 | **Univariable analysis:**  SBP$\leq$130 mmHg *vs* SBP>130 mmHg  For mRS$\leq$2: N=32 (66.7%) *vs* 17 (40.5%), **p=0.02**  **Multivariable analysis (multivariable ordinal logistic regression):**  **Mean SBP>130 mmHg:** **OR=2.66 (1.11–6.41)**  No significant results for the other parameters (24h mean SBP, percent reduction >15%) | HTF: HI-1, HI-2, PH-1, PH-2.  No clear definition of sICH | Not mentioned | HI: N=7 (7.8%), PH: N=16 (17.8%)  **24h mean SBP<=130 mmHg *vs* SBP>130 mmHg:** p=0.27  HI: 5 (10.4%) *vs* 2 (4.8)  PH: 6 (12.5%) *vs* 10 (23.8%) |
| Anadani et al, 2020^4^ | 0 | 1019 | **Univariable analysis:**  Good functional outcome (mRS: 0-2):  SBP<140 mmHg: 52%  SBP<160 mmHg: 52%  SBP<180 mmHg: 44%  **Inverse probability of treatment weights (IPTW)-adjusted multivariate analysis for SBP<140 mmHg:** associated with higher odds of good functional outcome compared to SBP<180 mmHg: OR=1.53 (1.07-2.19)  For patients with pre-treatment SBP$\geq$140 mmHg: Intensive (SBP<140 mmHg) and moderate (SBP<160 mmHg) BP targets had higher odds of good functional outcome than guideline-recommended BP targets: OR=1.75 (1.07-2.85) and OR=2.30 (1.17-4.52), respectively. | sICH defined as any intracranial hemorrhage within 72 hours associated with $\geq$4 points increase in the NIHSS or death following EVT. | Within 72h | sICH in intensive BP target: 3%  sICH in moderate BP target: 8%  sICH in guideline-recommended target: 5%  **On IPTW-adjusted multivariable analysis:** no significant association between BP targets and sICH  For patients with pre-treatment SBP$\geq$140 mmHg:  Intensive BP group had lower odds of sICH compared to guideline-recommended group: OR=0.38 (0.16-0.91) |
| **Studies without specific post-EVT BP targets** | | | | | | |
| Anadani et al,^14^ 2019 | 0 | 1361 | **rSBP and poor outcome:**  Model 1: OR=0.99 (0.98-0.99), **p=0.007**  Model 2: OR=0.97 (0.95-0.98), **p<0.001**  ***Subgroup analysis in patients with TICI 3:***  Model 1: OR=0.98 (0.97 to 0.99), **p=0.010**  Model 2: OR=0.98 (0.97 to 0.98), **p=0.014**  ***Subgroup analysis in patients with TICI 2B:***  Model 1: no significant results  Model 2: OR=0.99 (0.98 to 1.007)  SBPr included as a categorical variable (<1% (reference), 1%–10%, 11%–20%,>20%):  **1%–10% category:** OR=0.7; 95% CI 0.45 to 0.99  No significant results for >10% | Intraparenchymal, subarachnoid or intraventricular hemorrhage on post procedural CT associated with $\geq$4 NIHSS points during the first 72h after EVT | Within 72H | No significant association between rSBP and sICH |
| Anadani et al,^16^ 2019 | 4 | 1245 | **Univariable analysis:** *mRS 3-6 vs 0-2*  **Mean SBP:** 131 (16) vs 127 (14), **p<0.001**  **Maximum SBP:** 164 (25) vs 157 (24), **p<0.001**  **Minimum SBP:** 101 (18) vs 101 (15), p<0.999  **SBP SD:** 14 (5) vs 13 (5); **p=0.018**  **SBP Range:** 62 (26) vs 56 (24), **p<0.001**  **Multivariate analysis:**  **Mean SBP:** OR=0.86 (0.79–0.93), **p<0.001**  **Maximum SBP:** OR=0.9 (0.85–0.95), **p<0.001**  **SBP SD:** OR=0.99 (0.96–1.02), p=0.43  **SBP range:** OR=0.91 (0.86–0.96), **p=0.003**  No significant results for DBP parameters  SBP 101-120, 141-160 and >160 mmHg:  **SBP 141-160** and **>160 mmHg** had **43%** and **66%** lower odds of achieving good outcome at 90 days in comparison to 121-140 mmHg  mRS 0-2 rates were the highest for **101-120 mmHg** (**55%**) and the lowest for **>160 mmHg**. | sICH: any intraparenchymal, subarachnoid, intraventricular hemorrhage on post-procedural CT associated with $\geq$4 points on the NIHSS | Not mentioned but ECASS criteria | **Univariable analysis:** *no sICH vs sICH*  **Mean SBP:** 129 (15) vs 136 (15), **p<0.001**  **Maximum SBP:** 160 (24) vs 175 (27), **p<0.001**  **SBP SD:** 14 (5) vs 16 (7), **p=0.029**  **SBP range:** 59 (25) vs 71 (29), **p=0.002**  **Multivariate analysis:**  **Mean SBP:** OR=1.29 (1.19–1.5), **p=0.003**  **Maximum SBP:** OR=1.23 (1.2–1.3), **p<0.001**  **SBP SD:** OR=1.06 (1.01–1.13), **p=0.018**  **SBP range:** OR=1.19 (1.08–1.30), **p<0.001**  ***SBP as a categorial variable:***  **mean SBP** between **101-120 mmHg**: lower odds of sICH than the reference group: OR=0.37, **p=0.006**  For patients with SBP 141-160 and >160 mmHg: non-significant higher odds of sICH  The rate of sICH was the highest for the **SBP>160 mmHg** group (11%) *vs* **<100 mmHg** group (0%) |

**Abbreviations:** BP: blood pressure; BPV: BP variability; EVT: endovascular therapy; Nb: number; SBP: systolic blood pressure; DBP: diastolic blood pressure; IPTW: Inverse probability of treatment weights; MAP: mean arterial pressure; mRS: modified Rankin Scale; HTF: hemorrhagic transformation; ICH: intracranial hemorrhage; sICH: symptomatic intracranial hemorrhage

*Google scholar searched performed on May 3^rd^ 2020

**Supplemental Table IV. Definitions of intracranial and symptomatic intracranial hemorrhage**

| **Author, Year** | **Citation Nb^*^** | **Nb of patients** | **sICH definition** | **Timeframe for brain imaging** | **sICH results** |
| --- | --- | --- | --- | --- | --- |
| **Studies with specific post-EVT BP targets** | | | | | |
| Kim et al,^1^ 2019 | 8 | 211 | Intracranial hemorrhage (ICH) classified into 4 categories (HI-1, HI-2, PH-1, PH-2)  sICH: PH-2 combined with a neurologic deterioration of $\geq$4 points on the NIHSS from baseline | Median 1 day (IQR: 1-1) | HTF: N=88 (41.7%), HI-1: 11.4%, HI-2: 13.3%, PH-1: 6.1%, PH-2: 10.9%, sICH: N=20 (9.5%) |
| Chang et al,^3^ 2019 | 0 | 90 | ICH: HI-1, HI-2, PH-1, PH-2.  No clear definition of sICH | Not mentioned | HI: N=7 (7.8%), PH: N=16 (17.8%)  24h mean SBP $\leq$130 mmHg *vs.* >130 mmHg: no significant result in univariate analysis for HI or PH. |
| Anadani et al, 2020^4^ | 0 | 1019 | Any intracranial hemorrhage within 72 hours associated with $\geq$4 points increase in the NIHSS or death following EVT. | Within 72 hours | sICH in intensive BP target: 3%  sICH in moderate BP target: 8%  sICH in guideline-recommended target: 5%  **On IPTW-adjusted multivariable analysis:** no significant association between BP targets and sICH  For patients with pre-treatment SBP$\geq$140 mmHg:  Intensive BP group had lower odds of sICH compared to guideline-recommended group: OR=0.38 (0.16-0.91) |
| **Studies without specific post-EVT BP targets** | | | | | |
| Anadani et al,^14^ 2019 | 0 | 1361 | Intraparenchymal, subarachnoid or intraventricular hemorrhage on post procedural CT associated with $\geq$4 NIHSS points during the first 72h after EVT | Within 72 hours | sICH: N=70 (5%) |
| Anadani et al,^16^ 2019 | 4 | 1245 | sICH: any intraparenchymal, subarachnoid, intraventricular hemorrhage on post-procedural CT associated with $\geq$4 points on the NIHSS | Not mentioned but ECASS II criteria | sICH: N=58 (4.7%) |

**Abbreviations:** BP: blood pressure; BPV: BP variability; EVT: endovascular therapy; Nb: number; SBP: systolic blood pressure; DBP: diastolic blood pressure; MAP: mean arterial pressure; mRS: modified Rankin Scale; ICH: intracranial hemorrhage; sICH: symptomatic intracranial hemorrhage

*Google scholar searched performed on May 3^rd^ 2020

**Supplemental References:**

1. Kim TJ, Park HK, Kim JM, Lee JS, Park SH, Jeong HB, et al. Blood pressure variability and hemorrhagic transformation in patients with successful recanalization after endovascular recanalization therapy: A retrospective observational study. *Ann Neurol*. 2019;85:574-581

2. Zhang T, Wang X, Wen C, Zhou F, Gao S, Zhang X, et al. Effect of short-term blood pressure variability on functional outcome after intra-arterial treatment in acute stroke patients with large-vessel occlusion. *BMC Neurol*. 2019;19:228

3. Chang JY, Han MK. Postthrombectomy systolic blood pressure and clinical outcome among patients with successful recanalization. *Eur Neurol*. 2019;81:216-222

4. Anadani M, Arthur AS, Tsivgoulis G, Simpson KN, Alawieh A, Orabi Y, et al. Blood pressure goals and clinical outcomes after successful endovascular therapy: A multicenter study. *Ann Neurol*. 2020

5. Anadani M, Orabi Y, Alawieh A, Chatterjee A, Lena J, Al Kasab S, et al. Blood pressure and outcome post mechanical thrombectomy. *J Clin Neurosci*. 2019;62:94-99

6. McCarthy DJ, Ayodele M, Luther E, Sheinberg D, Bryant JP, Elwardany O, et al. Prolonged heightened blood pressure following mechanical thrombectomy for acute stroke is associated with worse outcomes. *Neurocrit Care*. 2019

7. Goyal N, Tsivgoulis G, Pandhi A, Dillard K, Alsbrook D, Chang JJ, et al. Blood pressure levels post mechanical thrombectomy and outcomes in non-recanalized large vessel occlusion patients. *J Neurointerv Surg*. 2018;10:925-931

8. Goyal N, Tsivgoulis G, Pandhi A, Chang JJ, Dillard K, Ishfaq MF, et al. Blood pressure levels post mechanical thrombectomy and outcomes in large vessel occlusion strokes. *Neurology*. 2017;89:540-547

9. Chu HJ, Lin CH, Chen CH, Hwang YT, Lee M, Lee CW, et al. Effect of blood pressure parameters on functional independence in patients with acute ischemic stroke in the first 6 hours after endovascular thrombectomy. *J Neurointerv Surg*. 2019

10. Cho BH, Kim JT, Lee JS, Park MS, Kang KW, Choi KH, et al. Associations of various blood pressure parameters with functional outcomes after endovascular thrombectomy in acute ischaemic stroke. *Eur J Neurol*. 2019;26:1019-1027

11. Chang JY, Jeon SB, Lee JH, Kwon OK, Han MK. The relationship between blood pressure variability, recanalization degree, and clinical outcome in large vessel occlusive stroke after an intra-arterial thrombectomy. *Cerebrovasc Dis*. 2018;46:279-286

12. Mistry EA, Sucharew H, Mistry AM, Mehta T, Arora N, Starosciak AK, et al. Blood pressure after endovascular therapy for ischemic stroke (best): A multicenter prospective cohort study. *Stroke*. 2019;50:3449-3455

13. Mistry EA, Mehta T, Mistry A, Arora N, Starosciak AK, De Los Rios La Rosa F, et al. Blood pressure variability and neurologic outcome after endovascular thrombectomy: A secondary analysis of the best study. *Stroke*. 2019:Strokeaha119027549

14. Anadani M, Arthur AS, Alawieh A, Orabi Y, Alexandrov A, Goyal N, et al. Blood pressure reduction and outcome after endovascular therapy with successful reperfusion: A multicenter study. *J Neurointerv Surg*. 2019

15. Mistry EA, Mistry AM, Nakawah MO, Khattar NK, Fortuny EM, Cruz AS, et al. Systolic blood pressure within 24 hours after thrombectomy for acute ischemic stroke correlates with outcome. *J Am Heart Assoc*. 2017;6

16. Anadani M, Orabi MY, Alawieh A, Goyal N, Alexandrov AV, Petersen N, et al. Blood pressure and outcome after mechanical thrombectomy with successful revascularization. *Stroke*. 2019;50:2448-2454

17. Cernik D, Sanak D, Divisova P, Kocher M, Cihlar F, Zapletalova J, et al. Impact of blood pressure levels within first 24 hours after mechanical thrombectomy on clinical outcome in acute ischemic stroke patients. *J Neurointerv Surg*. 2019;11:735-739

18. Matusevicius M, Cooray C, Bottai M, Mazya M, Tsivgoulis G, Nunes AP, et al. Blood pressure after endovascular thrombectomy: Modeling for outcomes based on recanalization status. *Stroke*. 2019:Strokeaha119026914
